# Psychological Capital on College Teachers’ and Students’ Entrepreneurial Performance and Sports Morality Under Social and Political Education

**DOI:** 10.3389/fpsyg.2022.810626

**Published:** 2022-03-30

**Authors:** Tao Lyu, Lijun Tang, Zeyun Yang

**Affiliations:** ^1^Department of Physical Education, Hohai University, Nanjing, China; ^2^Institute of Physical Education, Woosuk University, Wanju-gun, South Korea; ^3^Institute of Physical Education, Shanghai Normal University, Shanghai, China

**Keywords:** psychological capital appreciation, political and ideological education, entrepreneurial performance, sports morality, multiple linear regression

## Abstract

The aim of this study was to improve the entrepreneurial performance (EP) and sports morality of college teacher-and-student entrepreneurs (i.e., college entrepreneurs). Consequently, psychological capital (PsyCap) is creatively combined with social and political education (SPE) to explore college entrepreneurs’ EP and sports morality. First, following a theoretical model implementation, this article proposes several hypotheses. Then, a questionnaire survey (QS) was designed, and the data were analyzed. The results show that (1) gender has little impact on EP and sports morality; (2) PsyCap significantly affects the EP of college entrepreneurs at the age of 33–38 years; (3) in terms of educational background, average scores of PsyCap + SPE of bachelors are the highest, followed by masters or above, and finally, the college undergraduate; (4) the average score of PsyCap + SPE of married respondents is 4.0, while that of the unmarried is 3.7; (5) there is a significant difference between college entrepreneurs’ EP under the dimension of the basic enterprise situation; and (6) the average score of the length of entrepreneurship is 9.87, which has the most significant impact on the EP and sports morality, and the significance of sports morality is 0.04. Among them, the most significant impact on EP and sports morality is weekly sports participation, with a score of 9.67. Therefore, PsyCap + SPE plays a positive role in the EP of college entrepreneurs. In contrast, sports morality has little impact on EP. This study provides a reference for the research on the influence of entrepreneurship and sports morality of college entrepreneurs.

## Introduction

At present, as the main driving force for innovation to promote national development, entrepreneurial performance (EP) is often identified as the central concern of Innovation and Entrepreneurship Education (IEE) and entrepreneurship research. Initial entrepreneurial conditions significantly impact newly established enterprises (Victor, 2021). The enterprise can survive or grow better if the enterprise resource is rich at start-up. Of these, material capital, as a public resource, is particularly important to determine which college teacher and student entrepreneurs (hereafter referred to as college entrepreneurs) might lack prominent advantages over other entrepreneurs (Peng et al., 2020). Meanwhile, entrepreneurial practices have shown that entrepreneurs play a leading role in the initial and determinant enterprise development stages. Their innovation behaviors often involve high risks and are costly and rewarding (Khalid, 2020). Therefore, successful entrepreneurship often holds high requirements for entrepreneurs’ psychological quality. As a result, an increasing number of people pay attention to these special groups’ psychological and social capital. In particular, extensive research has been conducted on the EP of college entrepreneurs from the perspective of psychological capital (PsyCap).

Psychological capital was first proposed by Fred Luthans, a famous American scholar, in 2004 and extended to the Human Resource Management System (HRMS) (Luthans et al., 2004). He put forward four dimensions of PsyCap, namely, hope, efficiency, tenacity, and optimization, thereby laying the theoretical foundation of many subsequent studies in the field of psychology (Barratt and Duran, 2021; Gomes et al., 2021; Lu D. et al., 2021). The conceptual theory of PsyCap can improve the overall EP of college entrepreneurs through development and management proposed by Fred Luthans, who emphasizes the positive psychological power of individuals. Specifically, PsyCap mainly focuses on “who you are” or “what kind of person you want to be.” PsyCap is derived from positive organizational behavior, focusing on self-confidence, hope, tenacity, and optimism. Pandey et al. (2021) surveyed 309 employees from different industries in India, finding a positive correlation between inner entrepreneurial spirit, PsyCap, and work input. PsyCap partially regulated the relationship between inner entrepreneurial spirit and work input. Meanwhile, PsyCap could improve entrepreneurs’ EP by managing employees’ psychological ability (Pandey et al., 2021). Therefore, PsyCap is a subset of the individual’s positive psychological resources, covering self-confidence, a quasi-psychological state with both stable and unstable quality.

The objective of college social and political education (SPE) is to improve the social, political, and moral qualities of teachers and students through certain social concepts, political views, and ethical norms education (Du et al., 2020; Li et al., 2020; Wu A. et al., 2020). College SPE has a particular practical significance by providing solid support for the critical force of China’s economic construction, college entrepreneurs, IEE, and sports (Mu et al., 2020; Philpot et al., 2021).

At present, due to the poor entrepreneurial environment and lame EP, many domestic entrepreneurial enterprises are on the brink of bankruptcy. College entrepreneurs are one of the fundamental forces of entrepreneurship, and under such socio-economic background, the main problem to promote successful entrepreneurship has become improving their EP (Qian et al., 2018; Bronikowska et al., 2020; Wu Y. J. et al., 2020; Zhang Q. et al., 2020). In particular, EP is a crucial enterprise-strength index that reflects how well an enterprise completes a task or achieves a goal in entrepreneurship. Wu et al. (2018) revealed the impact of different entrepreneurial methods on entrepreneurship (Wu et al., 2018). The main influencing factors of EP include entrepreneurial team, entrepreneurs, and entrepreneurial environment. Studies have shown that PsyCap positively impacts EP (Ahmet et al., 2021).

Social and Political Education (SPE) accounts for a considerable proportion of the college curriculum in China, so SPE is introduced to explore the EP of college entrepreneurs. In contrast, physical exercise is closely related to individual psychological states, so this research also examines college entrepreneurs’ sports morality, which is the moral behavior of college entrepreneurs in sports activities (Wang et al., 2019; Wu W. et al., 2019; Rath et al., 2020). Most importantly, college physical education should stress the importance of effective in-classroom communication with students, as Wu Y. et al. (2019) revealed the importance of classroom communication with students in the college classroom (Wu Y. et al., 2019). Moreover, the core requirement of sports morality is respect and confidence, which is consistent with PsyCap + SPE.

To sum up, this study mainly investigates the impact of PsyCap on EP. At the same time, two essential factors in the studies and life of college entrepreneurs have been supplemented, namely, SPE and sports morality. In addition, this article also examines the impact of PsyCap + SPE on college entrepreneurs’ sports morality. As one of the hot fields in psychology, the research of PsyCap is vital. Combined with the current employment situation of college entrepreneurs, the research on the relationship between PsyCap and EP has an important practical significance. Accordingly, this study proposes all the hypotheses based on these two problems. The article framework is dissected as follows. First, the literature method is used to sort out and introduce the research status of EP and sports morality of college entrepreneurs. Second, the theoretical model is implemented, and research hypotheses are put forward. Based on this, the questionnaire survey (QS) is designed using the Analysis of Moment Structures (AMOS) 24.0 and Statistical Product and Service Solutions (SPSS) 25.0 software. Finally, the hypothesis is verified using the multiple linear regression (MLR) method. In previous studies, there are few studies on EP under the SPE of college entrepreneurs (Ratten and Usmanij, 2021). Therefore, the innovation of this article is to combine PsyCap and SPE to study the impact of their joint action on EP and sports morality of college entrepreneurs. The aim was to explore the relationship between the PsyCap of college entrepreneurs and their performance and to put forward some countermeasures. The research findings expand the research on the influencing factors of college entrepreneurs’ EP and sports morality.

### Determination of Research Indexes on Entrepreneurial Performance and Sports Morality of College Entrepreneurs

Financial indexes are more intuitive to measure enterprise performance and are easier to obtain. Enterprise financial statements can provide information, such as sales growth rate (SGR), return on investment (ROI), return on assets (ROA), and market price per share (Wu W. et al., 2020; Ratten and Usmanij, 2021; Subedi, 2021). However, specific enterprise situations, such as entrepreneurial efficacy and employee turnover rate (ETR), directly impact relevant entrepreneurial activities, so financial indexes should be adjusted according to the actual situations. Enterprise performance indexes can also be divided into absolute and relative indexes. The financial performance of start-ups is measured by the profit index relative to competitors (Hernández-Perlines et al., 2021). Although the relative performance index cannot effectively reflect the essence of entrepreneurship, comparing enterprises in different industries can reduce some problems. Meanwhile, objective indexes are quantifiable to evaluate EP (Ignatov, 2020). In contrast, subjective indexes are the personal evaluation of entrepreneurial efficiency and effect, which use scales to measure personal feelings toward EP, like the Likert-5-Grade Scale (Cid et al., 2019; Hasan et al., 2019). Objective indexes may not get accurate results because they involve enterprise confidentiality. Thus, subjective indexes are easier to measure EP (Adel, 2021; Lortie et al., 2021), including personal efficacy, employee satisfaction, and comparison with competitors. In summary, all methods can reflect the essential characteristics of entrepreneurship by measuring EP.

In addition, this section measures EP based on the enterprise’s financial indexes, such as the enterprise’s ROA, SGR, and ROI (Rogoza et al., 2018; Chen, 2019; Wu and Song, 2019). It also examines some subjective indexes, including enterprise ETR and entrepreneurial self-efficacy (ESE) (Yin et al., 2020; Zhang B. et al., 2020). EP can be divided into survival performance and growth performance in the growth process of start-ups (Chen and Yu, 2020). New enterprises are relatively simple and have a few personnel. Thus, the top priority is survival among the fierce industrial competition; only then may they expand or develop (Balasubramanian et al., 2020). Therefore, measuring EP from its survival and growth is essential. Based on the division of enterprise growth, survival performance and growth performance are selected as the measurement indexes of EP of college entrepreneurs (Yi, 2021). The research indexes of sports morality of college entrepreneurs mainly focus on the moral requirements at the individual level (Lu G. et al., 2021). Later, sports morality is divided into two dimensions, namely, sports quality and sportsmanship, based on previous experience. According to the previous research on PsyCap + SPE, the fusion indexes include PsyCap (i.e., self-confidence, optimism, tenacity, and hope) and SPE (i.e., self-esteem and self-transcendence).

### Model Implementation on Entrepreneurial Performance and Sports Morality of College Entrepreneurs

As a positive psychological state, PsyCap significantly impacts the EP of different individuals or organizations. Many scholars have found a significant positive correlation between PsyCap and various elements of EP (Luo et al., 2021). Gao et al. (2021) expounded on the relationship between the college entrepreneurs’ PsyCap and its EP. They concluded that PsyCap significantly impacted EP (Gao et al., 2021). Grözinger et al. (2021) reasoned that PsyCap was an essential psychological resource and analyzed it using a structural equation model. The results showed that PsyCap had a significant positive impact on EP (Grözinger et al., 2021). In short, PsyCap plays a positive role in EP according to a relevant literature review (Digan et al., 2019). Tang (2020) proved the positive correlation between PsyCap dimensions and EP (Tang, 2020). Bockorny and Youssef-Morgan (2019) reached the same conclusion through empirical research on employees and leaders: PsyCap and its elements were significantly positively correlated with performance (Bockorny and Youssef-Morgan, 2019). Combing relevant theories of PsyCap indicates that PsyCap is a positive psychological state in the process of individual growth and self-development. Good PsyCap helps improve entrepreneurs’ social ability and improve their EP.

**FIGURE 1:**
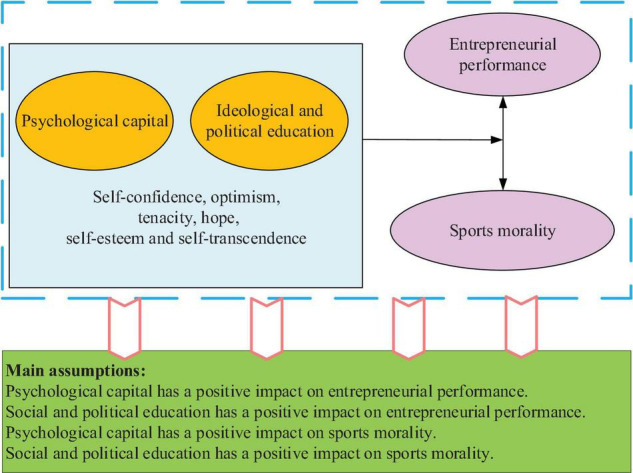
Model of the influence of psychological capital plus social and political education (PsyCap + SPE) on the entrepreneurial performance (EP) and sports morality of college entrepreneurs.

Based on the theoretical research of PsyCap, SPE, EP, and sports morality, a theoretical model is implemented concerning the impact of PsyCap + SPE on the EP and sports morality of college entrepreneurs (Gorostiaga et al., 2019). Figure 1 shows the theoretical model of PsyCap + SPE’s influence on EP and sports morality of college entrepreneurs under SPE.

According to Figure 1, the research model consists of three aspects, namely, research level, influencing factors, and research path. The six PsyCap + SPE indexes are independent variables, and the four dimensions of EP and sports morality are dependent variables. The control variables of the model are the basic personal information of college entrepreneurs, including age, gender, marital status, education level, sports participation, and entrepreneurship experience. Figure 1 manifests the main influencing factors and hypothesis of PsyCap + SPE, and the specific hypotheses will be given in the following section.

### Proposal of the Hypothesis

The PsyCap theory emphasizes on an individual’s positive psychological power. PsyCap is derived from positive organizational behavior. Positive organizational behavior improves work performance by applying helpful human resources and positive, measurable, and manageable psychological factors (Breznitz and Zhang, 2020). Positive psychological states, such as self-confidence, hope, tenacity, and optimism, have become the leading research objects of positive organizational behavior because they have formed universal quantifiable standards (Alshebami et al., 2020). Therefore, academic circles agree that PsyCap is mainly composed of four dimensions, namely, self-confidence, hope, tenacity, and optimism. Besides, the positive organizational behavior-based PsyCap and abilities of college entrepreneurs can also improve their EP.

Among them, self-confidence is the belief that college entrepreneurs can adjust their cognitive pattern and achieve their work goals (Huang et al., 2021). There is an internal positive correlation between employees’ self-confidence and job performance. Meanwhile, self-confidence can stimulate the external related factors of organizations and influence job performance (Choi and Markham, 2019). In positive psychology, hope is an individual’s overall perception that goals are attainable with beliefs and efforts, and they have the pathways to go after the desired goals. Tenacity refers to an individual’s ability to quickly recover from adversity, conflict, and failure (Alfalih and Ragmoun, 2020). Strong willpower can help employees overcome work difficulties and improve their ability to learn new knowledge, improving their work performance. Optimism is a positive attribution tendency. It can make people consider some favorable situations as lasting and universal and some negative ones as temporary. PsyCap helps to improve entrepreneurs’ social and communication skills, which is also very helpful for entrepreneurs to strengthen their EP (Shi et al., 2020). Based on the above theoretical analysis, it is concluded that entrepreneurs with positive PsyCap have significant psychological advantages in determining entrepreneurial goals, exploring entrepreneurial opportunities, overcoming various entrepreneurial difficulties, and inspiring the entrepreneurial potential of the organization (Jena, 2020). Such psychological advantages can promote entrepreneurs’ entrepreneurial activities and improve their EP.

Thus, PsyCap plays a positive role in the EP of college entrepreneurs. From the perspective of SPE, self-esteem positively affects employee performance; besides, previous studies have shown that enterprise performance will be improved when employees have an intention to transcend themselves (Grimaldi et al., 2019; Sánchez-Sáez et al., 2020). Furthermore, the four dimensions of PsyCap, namely, self-confidence, optimism, tenacity, and hope, play an essential role in constructing sports morality. In comparison, SPE cultivates students’ self-esteem and self-transcendence, both essential in sports moralities (Raquel et al., 2021). Because the four dimensions of PsyCap and the two dimensions of sports morality belong to a fundamental human character, it is of little significance to study their relationship. Therefore, this article mainly explores the impact of joint action of PsyCap + SPE on EP and sports morality. Subsequently, 24 hypotheses about PsyCap and sports morality are put forward and are listed in Table 1.

**TABLE 1:** 24 Hypotheses about influencing factors of psychological capital (PsyCap) and sports morality.

Hypothesis number	Contents of hypotheses
H1	Self-confidence has a positive effect on survival performance
H2	Optimism has a positive effect on survival performance
H3	Tenacity has a positive effect on survival performance
H4	Hope has a positive effect on survival performance
H5	Self-esteem has a positive effect on survival performance
H6	Self-transcendence has a positive effect on survival performance
H7	Self-confidence has a positive effect on growth performance
H8	Optimism has a positive effect on growth performance
H9	Tenacity has a positive effect on growth performance
H10	Hope has a positive effect on growth performance
H11	Self-esteem has a positive effect on growth performance
H12	Self-transcendence has a positive effect on growth performance
H13	Self-confidence has a positive effect on sports quality
H14	Optimism has a positive effect on sports quality
H15	Tenacity has a positive effect on sports quality
H16	Hope has a positive effect on sports quality
H17	Self-esteem has a positive effect on sports quality
H18	Self-transcendence has a positive effect on sports quality
H19	Self-confidence has a positive effect on sportsmanship
H20	Optimism has a positive effect on sportsmanship
H21	Tenacity has a positive effect on sportsmanship
H22	Hope has a positive effect on sportsmanship
H23	Self-esteem has a positive effect on sportsmanship
H24	Self-transcendence has a positive effect on sportsmanship

After the hypothesis is put forward, the QS are designed. Then, the reliability and validity of the QS are tested. Finally, the QS results are analyzed using MLR to verify the hypothesis. The data analysis tools include SPSS 25.0 and AMOS 24.0.

### Design of the Questionnaire Survey

Psychological capital falls into four dimensions, namely, self-confidence, hope, tenacity, and optimism. Among them, self-confidence refers to the belief that one adjusts his cognitive model and takes action to achieve his work goals. Hope is an individual’s overall perception that dreams are attainable with beliefs and efforts, and they have the pathways to go after the desired goals. Tenacity is an individual’s ability to quickly recover from adversity, conflict, and failure (Hatak et al., 2021). Optimism is a positive attribution tendency, and it regards some favorable situations as lasting and universal and some adverse events as temporary (Brixiová et al., 2020). Based on the above analysis, the QS is divided into three parts, namely, the QS for PsyCap + SPE, the QS for the EP of college entrepreneurs, and the QS for sports morality. Table 2 shows the core issues of the QS for PsyCap + SPE.

**TABLE 2:** The core issues of the designed questionnaire survey (QS) for PsyCap + social and political education (SPE).

Index of measurements	Specific contents
Self-confidence	I believe I can contribute to the group
	I believe I can achieve the goal
	I believe I can analyze issues in the long term
Optimism	I am always positive about my future
	I will positively face whatever difficulties
	I have all I wished
Tenacity	I can stay calm under pressure
	I believe I can overcome all the difficulties
	I can stand up from where I fall
Hope	I believe there are always ways to solve troubles
	I believe tomorrow will be better once I try
	Against difficulties, I will actively seek solutions
Self-esteem	I think I have never done anything against morality
	I think I always communicate with others in a friendly way
Self-transcendence	I often think about the current situation and find ways to improve
	I can learn through adversities conquering

Table 2 shows that the core issues of PsyCap + SPE comprise six levels, namely, self-confidence, optimism, tenacity, hope, self-esteem, and self-transcendence, with a total of 16 specific core issues.

The second part of the QS includes core issues of the QS for the EP of college entrepreneurs, as shown in Table 3.

**TABLE 3:** Core issues of the QS for the entrepreneurial performance (EP) of college entrepreneurs.

Indexes of measurements	Specific contents
Survival performance	In the face of a crisis, my company can respond positively
	Our company can run for more than 5 years
Growth performance	The annual profit growth rate of my company exceeds that of competitors
	The annual growth rate of employees
	The annual growth rate of total sales

Table 3 shows that the QS for EP of college entrepreneurs is composed of survival and growth performance, containing five specific issues.

Table 4 lists the core issues in QS for sports morality of college entrepreneurs.

**TABLE 4:** Core issues of QS for sports morality of college entrepreneurs.

Indexes of measurements	Specific contents
Sports quality	I abide norms and requirements of sports
	I take sports to exercise actively
	I respect the sports habits of others
Sportsmanship	I do not have conflicts with others
	I will give instant help once noticing someone gets hurt

Table 4 demonstrates the core issues of the QS for sports morality of college entrepreneurs, including sports quality and sportsmanship, with five specific indicators.

The QS respondents are college entrepreneurs from five college entrepreneurship bases in a city, and 240 QSs are distributed. The QS is distributed online and offline simultaneously; 120 copies are sent online through email, and 120 copies are distributed offline to college entrepreneurs of the college entrepreneurship base. All QSs are completed anonymously. A five-point system scores the QS topics, namely, totally disagree, disagree, neutral, agree, and totally agree. A total of 223 QSs were collected, with a recovery rate of 92.9%, an effective rate of 87.5%, and 210 valid QSs.

Analysis of Moment Structures (AMOS) 23.0 and SPSS 25.0 analyze the QS data. Based on a descriptive statistical analysis, the reliability and validity of each variable are tested by Cronbach’s alpha coefficient and confirmatory factor analysis (CFA). Then, correlation regression analyses are conducted, focusing on the relationship between PsyCap, SPE, and EP of college entrepreneurs. The descriptive analysis is used to analyze the basic personal information data in the QSs. The reliability and validity analysis is used to measure data consistency and reliability. The reliability analysis of the data is carried out based on the literature method by Khalid et al. (2020). Equation 1 calculates the Cronbach’s alpha coefficient. If the coefficient is more significant than 0.8, the scale’s reliability is good.


(1) $$\alpha =\frac{\text{K}}{\text{K}-1}\,\left(1-\frac{\sum_{i=1}^{\text{K}}\sigma_{{\text{Y}}_{\text{i}}}^{2}}{\sigma_{\text{X}}^{2}}\right)$$

In Equation 1, *K* denotes the number of items in the QS, $\sigma_{X}^{2}$ indicates the variance of the total samples, and $\sigma_{\text{Y\_i}}^{2}$ is the variance of the observation samples.

The validity test adopts the Kaiser–Meyer–Olkin (KMO) and the Bartlett spherical test. If the KMO value exceeds 0.8, the data are acceptable (Khalid et al., 2020). Then, the model fit is tested based on the absolute fitting index (X^2^/df), root mean square error (RMR), goodness-of-fit index (GFI), root mean square error of approximation (RMSEA), and two relative fitting indexes [i.e., comparative fit index (CFI) and normed fit index (NFI)]. When X2/df is less than 3, the model fits better. The model is well fitted when GFI, CFI, and NFI are more than 0.9. When RMR and RMSEA are less than 0.08, the model is acceptable. Finally, the three parts of the QS are analyzed using the CFA. When the factor load is between 0.5 and 0.95, there is no significant error variance in the QS, and the effect is good (Yu et al., 2020; Zhao and Zhang, 2021).

Subsequently, the proposed hypotheses are verified using the MLR analysis, by constructing the regression model and then analyzing the model combined with particular problems (Dong et al., 2020). Equation 2 shows the model MLR equation.


(2) Fi=θ⁢1+θ2⁢M1+θ⁢M23+…+θn⁢Mn+1+δi

In Equation 2, θ_1_ is a constant item, *M* represents several dimensions of the scale, and θ_2_, θ_3_, and θ_n_ represent variable coefficients. *F*_*i*_ indicates the dependent variables of EP and sports morality. δ*_*i*_* signifies the residual item.

### Descriptive Analyses of Data in Questionnaire Survey

Table 5 describes college entrepreneurs’ basic information (e.g., age, gender, wedlock, and diploma). It also analyzes the entrepreneurial situation (e.g., length of enterprise operation, entrepreneurial motivation, and employee number) and sports participation (e.g., weekly physical exercises, interest in physical education, and ways to understand sports morality) (Ma et al., 2018). Table 5 details the statistical data.

**TABLE 5:** Descriptive analyses of QS data.

Basic personal information	Types	Number of objects
Age	Below 24	78
	25–32	42
	33–38	90
Gender	Female	103
	Male	107
Diploma	College undergraduate	31
	Bachelor	93
	Masters or above	86
Wedlock	Unmarried	84
	Married	126
Length of entrepreneurship	Within 2 years	46
	3–7 years	61
	8 years and above	103
Entrepreneurial motivation	Personal ideal	114
	To improve livelihood	65
	Following the trend	17
	Others	14
Number of employees	15 people and below	82
	16–60 people	34
	61–100 people	94
Physical exercise per week	Twice	45
	Three to four times	133
	Five times and above	32
Interest in physical education	Very interested	67
	Interested	109
	Not interested	34
Ways to understand sports morality	Class teaching in school	89
	Network	109
	Newspaper	12

Table 5 shows that the surveyed college entrepreneurs aged mostly 33–38 years, 43.3% of all age groups, followed by entrepreneurs aged below 24 years, and 20.0% of entrepreneurs aged 25–32 years. Such distribution may be because the entrepreneurs aged below 24 years and those aged 33–38 years have less pressure, while those aged 24–32 years have greater pressure in life. In fact, Apparently, gender distribution among entrepreneurs is symmetrical. From the perspective of educational background, entrepreneurs with bachelor’s degree, master’s degree, or above account for a relatively high proportion. Among them, bachelors’ degree holders account for the highest proportion (44.3%), followed by master’s degree or above, and the proportion of the college undergraduate is the lowest (14.8%), which is consistent with the increasing entrepreneurial market in China in recent years (Hernando et al., 2018; Huéscar et al., 2020). Married entrepreneurs take up 60.1% higher than unmarried ones, and such distribution is consistent with age distribution (Zhao et al., 2021). Meanwhile, the entrepreneurship length of 8 years and above accounts for the most (49.1%), followed by 3–7 years. The minimum entrepreneurship length is within 2 years, consistent with the age distribution (Rajah et al., 2021). The entrepreneurial motivations from most prominent to the least are “personal ideal” (54.3%), “to improve livelihood,” and “following the trend.” In a nutshell, most college entrepreneurs pay more attention to their spiritual needs, accounting for as high as 44.8% of 61–100 respondents. Such distribution is consistent with the age distribution and entrepreneurship length. Additionally, 63.3% of college entrepreneurs participate in physical exercise 3–4 times per week, 21.4% less than two times per week, and 15.2% more than five times; overall, they take physical exercise less frequently. Furthermore, 51.9% of college entrepreneurs have shown interest in college physical education, 31.9% say they are very interested, and 16.2% declare to not be interested; such a distribution is consistent with their weekly physical exercise distribution (Park et al., 2021). Finally, college entrepreneurs understand sports information mainly through the network, which also conforms to the characteristics of the Internet era.

### Reliability and Validity Analysis of Questionnaire Survey Data

**FIGURE 2:**
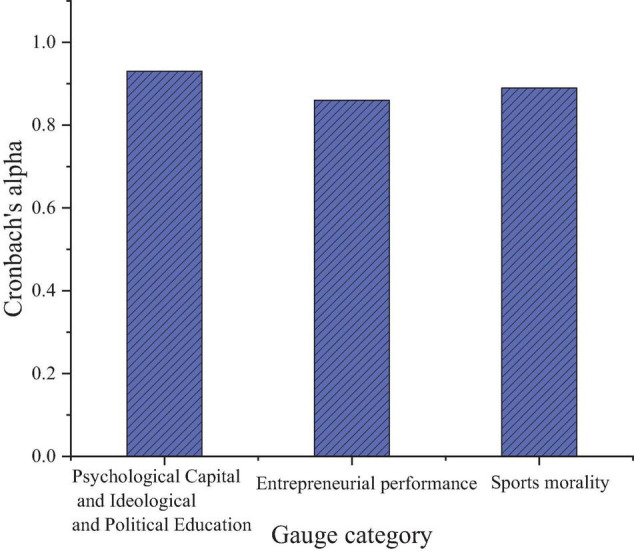
Questionnaire survey (QS) reliability analysis.

Figure 2 tests the reliability of the QS data through Cronbach’s alpha coefficient.

Figure 2 tells that the Cronbach’s coefficient of PsyCap + SPE, EP, and sports morality is more than 0.8, and the QS is reliable. The reliability is high, and the QS indexes are good, laying the foundation for the subsequent QS data analysis.

Table 6 shows the validity analyses results of the three parts of the QS.

**TABLE 6:** Questionnaire Survey validity analysis.

Sections of QS	Kaiser–Meyer–Olkin (KMO)	Chi-square test of Bartlett’s test	Significance
PsyCap + SPE	0.92	4,632.92	0.00
EP	0.81	1,747.54	0.00
Sports morality	0.87	1,871.76	0.00

As shown in Table 6, the KMO values of PsyCap + SPE, EP, and sports morality are all greater than 0.8, with statistical significance and promising validity. Thus, CFA can be carried out.

**FIGURE 3:**
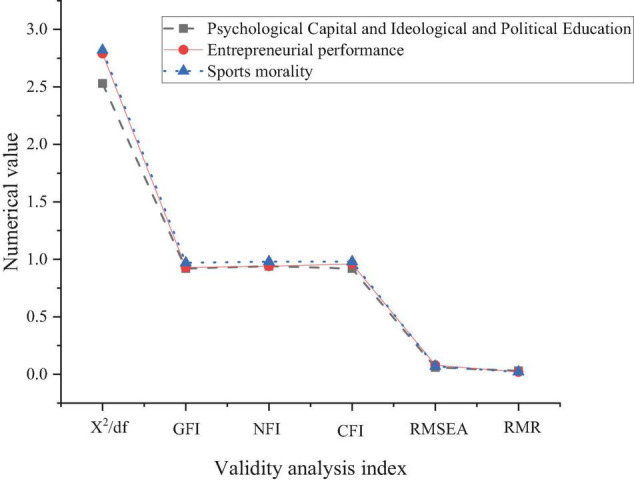
Model fitting index.

Figure 3 verifies the structural equation model by AMOS.

As shown in Figure 3, the X^2^/df indexes of PsyCap + SPE, EP, and sports morality are less than 3, indicating that the model fits well. The GFI, CFI, and NFI are greater than 0.9, proving that the model fits well. Meanwhile, RMR and RMSEA are less than 0.08, verifying the model’s validity. The above indexes demonstrate that the proposed model fits well, with high validity.

**FIGURE 4:**
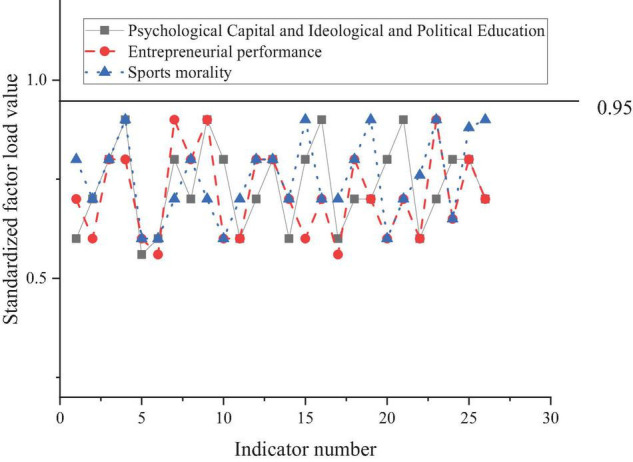
Results of confirmatory factor analysis (CFA).

Figure 4 shows 26 indexes of the PsyCap + SPE, EP, and sports moralities as 1–26 and analyzes them with CFA.

As shown in Figure 4, the standardized factor load of each index in the PsyCap + SPE, EP, and sports morality QS is statistically significant (between 0.5 and 0.95), so the overall validity of the QS is good.

### Influence of Basic Information on Psychological Capital + Social and Political Education, Entrepreneurial Performance, and Sports Morality

Table 7 shows the significant differences between college entrepreneurs of different ages.

**TABLE 7:** Significant difference of ages.

Categories of measurement	Ages	Average significance
PsyCap + SPE	Less than 24 years old	0.04
	25–32 years old	
	32–38 years old	
EP	Less than 24 years old	0.03
	25–32 years old	
	32–38 years old	
Sports morality	Less than 24 years old	0.06
	25–32 years old	
	32–38 years old	

**FIGURE 5:**
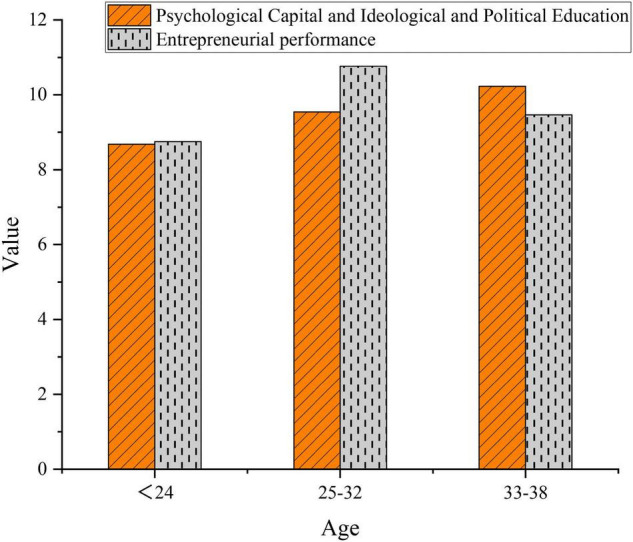
Average scores of college entrepreneurs of different ages.

As shown in Table 7, the statistical significance of sports morality is greater than 0.05, so college entrepreneurs of different ages show no difference in sports morality, which is consistent with the research results of Lu (2021). In comparison, there is a significant difference in PsyCap + SPE and EP between college entrepreneurs of different ages. Figure 5 calculates the average PsyCap + SPE and EP using the SPSS software.

As shown in Figure 5, entrepreneurs aged 33–38 years score the highest for average PsyCap + SPE score, followed by 25–32 years old, and finally, below 24 years old, which is consistent with the age distribution in the descriptive analysis. EP is the highest among entrepreneurs aged 25–32 years, followed by 33–38 years old, and the lowest among entrepreneurs below 24 years old. Possibly, it is because college entrepreneurs have the most entrepreneurial vitality during the age of 25–32 years. According to the research by Xie et al. (2021), entrepreneurs aged 30–40 years have the most entrepreneurial vitality. Such a difference between the literature and this study may be due to the sample size (Xie et al., 2021).

Table 8 analyzes the differences of college entrepreneurs of different genders in PsyCap + SPE, EP, and sports morality.

**TABLE 8:** Statistical differences in gender.

Categories of measurement	Gender	Average significance
PsyCap + SPE	Female	0.06
	Male	
EP	Female	0.06
	Male	
Sports morality	Female	0.07
	Male	

Table 8 suggests that the significance of PsyCap + SPE, EP, and sports morality is greater than 0.05. Thus, there is no statistical difference between college entrepreneurs of different genders in PsyCap, SPE, EP, and sports morality.

Table 9 shows college entrepreneurs’ differences in PsyCap + SPE, EP, and sports morality with different educational backgrounds.

**TABLE 9:** Prominent differences of different diplomas.

Categories of measurement	Diploma	Average significance
PsyCap + SPE	College undergraduate	0.04
	Bachelor	
	Masters or above	
EP	College undergraduate	0.06
	Bachelor	
	Masters or above	
Sports morality	College undergraduate	0.06
	Bachelor	
	Masters or above	

**FIGURE 6:**
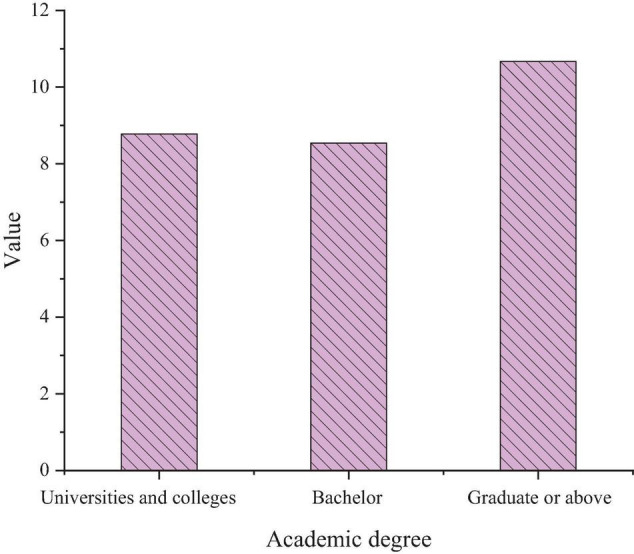
Average PsyCap scores of college entrepreneurs with different educational backgrounds.

Table 9 implies that college entrepreneurs with different educational backgrounds show significant differences in PsyCap + SPE and less difference in EP and sports morality. Figure 6 calculates the average PsyCap + SPE score using SPSS.

Figure 6 indicates that the PsyCap + SPE scores of “masters or above” are the highest, followed by “bachelor,” and finally, “college undergraduate.” Since “masters or above” entrepreneurs receive more psychological education, the score is higher, and “bachelors” is more stressful than “college undergraduates,” so the score is the least.

Table 10 shows the differences of college entrepreneurs’ PsyCap + SPE, EP, and sports morality in the wedlock dimension.

**TABLE 10:** Significant differences in wedlock dimension.

Categories of measurement	Wedlock	Average Significance
PsyCap + SPE	Married	0.04
	Unmarried	
EP	Married	0.06
	Unmarried	
Sports morality	Married	0.06
	Unmarried	

Table 10 demonstrates that the significance of EP and sports morality is greater than 0.05. Thus, college entrepreneurs with different marital statuses present no significant difference in EP and sports morality but PsyCap + SPE. More precisely, the average PsyCap + SPE score of married respondents is 4.0, which is higher than 3.7 of the unmarried respondents.

Table 11 shows the significance analyses of the basic situation of enterprises.

**TABLE 11:** College entrepreneurs’ significant difference analysis from the enterprise situation.

Categories of measurement	Basic enterprise situation	Average significance
PsyCap + SPE	Length of entrepreneurship	0.07
	Entrepreneurial motivation	
	Number of employees	
EP	Length of entrepreneurship	0.04
	Entrepreneurial motivation	
	Number of employees	
Sports morality	Length of entrepreneurship	0.08
	Entrepreneurial motivation	
	Number of employees	

Table 11 reveals that college entrepreneurs show a significant difference in EP under the dimension of basic enterprise situation. The length of entrepreneurship has the largest impact on EP, with an average score of 9.87.

Table 12 shows the college entrepreneurs’ statistical difference under the dimension of sports participation.

**TABLE 12:** College entrepreneurs’ statistical difference under sports participation dimension.

Categories of measurement	Sports participation	Average significance
PsyCap + SPE	Weekly sports participation	0.07
	Interest in physical education in college	
	Related ways to know about sports morality	
EP	Weekly sports participation	0.09
	Interest in physical education in college	
	Related ways to know about sports morality	
Sports morality	Weekly sports participation	0.04
	Interest in physical education in college	
	Related ways to know about sports morality	

Table 12 reflects that the significance of college entrepreneurs’ PsyCap + SPE and EP is greater than 0.05, indicating that the basic sports participation is not closely related to these two aspects. In comparison, college entrepreneurs’ sports morality under the sports participation dimension is statistically significant with 0.04, and the greatest impact is the “weekly sports participation,” with 9.67.

### Verification of the Hypotheses

Subsequently, SPSS 25.0 performs a binary analysis on PsyCap + SPE, EP, and sports morality correlation. The analysis results show that the variables proposed are highly positively correlated, so the proposed hypotheses hold. Based on this, Equation 2 is used for the MLR analysis, and the hypothesis verification results are tabulated in Table 13.

**TABLE 13:** Verification on the hypothesis about PsyCap and sports morality.

Hypotheses		True or false
H1	Self-confidence has a positive effect on survival performance	True
H2	Optimism has a positive effect on survival performance	True
H3	Tenacity has a positive effect on survival performance	True
H4	Hope has a positive effect on survival performance	True
H5	Self-esteem has a positive effect on survival performance	False
H6	Self-transcendence has a positive effect on survival performance	False
H7	Self-confidence has a positive effect on growth performance	True
H8	Optimism has a positive effect on growth performance	True
H9	Tenacity has a positive effect on growth performance	True
H10	Hope has a positive effect on growth performance	True
H11	Self-esteem has a positive effect on growth performance	False
H12	Self-transcendence has a positive effect on growth performance	False
H13	Self-confidence has a positive effect on sports quality	True
H14	Optimism has a positive effect on sports quality	True
H15	Tenacity has a positive effect on sports quality	True
H16	Hope has a positive effect on sports quality	True
H17	Self-esteem has a positive effect on sports quality	True
H18	Self-transcendence has a positive effect on sports quality	True
H19	Self-confidence has a positive effect on sportsmanship	True
H20	Optimism has a positive effect on sportsmanship	True
H21	Tenacity has a positive effect on sportsmanship	True
H22	Hope has a positive effect on sportsmanship	True
H23	Self-esteem has a positive effect on sportsmanship	True
H24	Self-transcendence has a positive effect on sportsmanship	True

As shown in Table 13, four hypotheses do not hold, namely, (1) self-esteem has a positive effect on survival performance; (2) self-transcendence has a positive effect on survival performance; (3) self-esteem has a positive effect on growth performance; and (4) self-transcendence has a positive effect on growth performance. That is to say, growth performance is not affected by self-esteem and self-transcendence. Survival performance and growth performance are important influencing factors of EP, while self-esteem and self-transcendence are important representative indexes of sports morality. Therefore, sports morality does not directly positively impact EP. In terms of PsyCap, “self-confidence,” “optimism,” “tenacity,” and “hope” have a positive impact on EP. Maykrantz et al. (2021) believed that PsyCap had a positive effect on EP, which is consistent with the research results of this study.

## Discussion

The research topic of this article is the impact of PsyCap on EP, a field of Psychology. The relationship between the four concepts of PsyCap, namely, “self-confidence,” “optimism,” “tenacity,” and “hope,” and EP shows that PsyCap has a positive impact on EP, which is consistent with the conclusions of the literature related to PsyCap cited. Differently, this article starts with four specific concepts (Anglin et al., 2018). Mayr et al. (2021) researched the characteristics of entrepreneurs. They revealed that the features of entrepreneurs were related to entrepreneurial failure, and gender and management experience directly affected entrepreneurial success (Mayr et al., 2021). In comparison, this study makes a more specific analysis of the characteristics of entrepreneurs. There is no obvious relationship between college entrepreneurs under gender, age, sports participation, and basic enterprise situation difference. Meanwhile, Octavia et al. (2020) used a combination of directional and quantitative methods. They concluded that small and medium-sized enterprises’ (SMEs) performance was significantly related to the enterprise situation (Octavia et al., 2020). The result is consistent with this study. Differently, this article studies the influencing factors of EP. The final hypothesis test indicates that PsyCap positively impacts EP, while sports morality has minimal impact on EP. Such a conclusion is consistent with the research conclusion on the positive role of PsyCap in the latest research of Welter and Scrimpshire (2021).

## Conclusion

Based on the MLR method, this article creatively starts with the studies and life of college entrepreneurs and discusses the impact of the PsyCap + SPE on college entrepreneurs’ EP and sports morality. The main research topic of this article is the impact of PsyCap on EP. The innovation lies in combining PsyCap and SPE to study their combined effect on EP. The results corroborate that the gender of the surveyed college entrepreneurs has no impact on the PsyCap + SPE, EP, and sports morality. In comparison, age, educational background, marital status, basic enterprise situation, and sports participation impact college entrepreneurs’ PsyCap + SPE, EP, and sports morality. The theoretical significance of this study is that it adds the specific impact of the four basic concepts of PsyCap on EP. It provides a reference for improving EP in higher institutions at the practical level. Significantly, it gives a reference for developing PsyCap in the field of EP. The research significance in entrepreneurship is to put forward the improvement direction for the continuously optimizing entrepreneurial environment. Finally, the shortcomings are summarized. First, the impact of the basic enterprise situation on sports morality has not been studied by subdivided indexes (i.e., sports quality and sportsmanship), and the sample size is small. Second, the research objects are teachers and students of University Entrepreneurship Bases. The research results have a certain stability, so the reliability test of the experiment has not been carried out. Therefore, future research will conduct in-depth research on the QS design to obtain more refined indexes and to increase the sample size. Moreover, it is necessary to conduct in-depth reliability tests for the experiments. The research findings will lay a foundation for the wide application of PsyCap in the field of college entrepreneurs. The theoretical significance of this article is to provide more diversified directions for developing PsyCap in the field of EP, such as the combination with sports morality. The practical significance of this study lies in applying the MLR analysis in the field of Psychology and has achieved good results. The research findings will be applied to the college IEE to impact college IEE positively. Finally, the research findings provide a reference for combining psychology and entrepreneurship, conducive to further applying psychology in solving practical problems.

## Data Availability Statement

The raw data supporting the conclusions of this article will be made available by the authors, without undue reservation.

## Ethics Statement

The studies involving human participants were reviewed and approved by Shanghai Normal University. The patients/participants provided their written informed consent to participate in this study. Written informed consent was obtained from the individual(s) for the publication of any potentially identifiable images or data included in this article.

## Author Contributions

All authors listed have made a substantial, direct, and intellectual contribution to the work, and approved it for publication.

## Conflict of Interest

The authors declare that the research was conducted in the absence of any commercial or financial relationships that could be construed as a potential conflict of interest.

## Publisher’s Note

All claims expressed in this article are solely those of the authors and do not necessarily represent those of their affiliated organizations, or those of the publisher, the editors and the reviewers. Any product that may be evaluated in this article, or claim that may be made by its manufacturer, is not guaranteed or endorsed by the publisher.
